# Chemical and Bioactive Profiling, and Biological Activities of Coral Fungi from Northwestern Himalayas

**DOI:** 10.1038/srep46570

**Published:** 2017-04-19

**Authors:** Sapan Kumar Sharma, Nandini Gautam

**Affiliations:** 1 Mushroom Research & Training Centre, Department of Plant Pathology, CSK, Himachal Pradesh Agriculture University, Palampur, 176 062, India; 2 Centre for Environment Science and Technology, School of Environmental and Earth Sciences, Central University of Punjab Bathinda, 151 001, India

## Abstract

*Ramaria* Fr. and *Clavaria* L. are the two major genera of coral mushrooms within families Gomphaceae and Clavariaceae, respectively. Besides having important role in forest ecology, some species of these are reported to possess high nutraceutical and bioactive potential. There is a hidden diversity of coral mushrooms in Northwestern Himalayas. Present studies describe the detailed biochemical profiling and antioxidant, and antibacterial activities of twelve coral mushroom species. Biochemical profiling of nutrients and nutraceuticals was done with standard techniques and by using HPLC, UPLC and GC. Experiments were also conducted to check the toxic metals detection. Antioxidant activities were calculated using EC50 values from mushroom extracts. Antibacterial activities were checked on six pathogenic bacterial strains through minimum inhibition concenterations. Although, differences were observed in the net values of individual species but all the species were found to be rich in protein, macro and micro minerals, carbohydrates, unsaturated fatty acids, essential amino acids, phenolics, tocopherols, anthocynadins and carotenoids. All the species showed significant antioxidant and antibacterial activities. These species are reported to free from heavy toxic metals. Present studies will open the way for their large scale commercial exploitations and use in pharmaceutical industries as antioxidant, antibacterial and nutraceutical constituents.

Wild mushrooms are used from ancient times and have a long history as nutritious tasty food items with low calorific values and high in proteins, vitamins, iron, zinc, selenium, sodium, chitin, fibres and minerals^1^,^2^,^3^. Besides high nutritional value these have well known medicinal utility due to well established therapeutic potential^4^,^5^,^6^,^7^,^8^,^9^,^10^. Fruitbodies and mycelium of some species are reported to contain important bioactive commpounds with high antioxidant potential^11^. Extracts from the wild fungi are considered as important remedies for the prevention and cure of many diseases from decades in several parts of the world^12^,^13^. Immunomodulating molecules extracted from mushrooms are used nowadays to improve immune function in cancer patients during radio and chemotherapy and help to prolong survival times in many type of cancers^14^. Role of the wild edible mushrooms in lowering blood pressure and free cholesterol in plasma is well understood^15^. The bioactive compounds extracted from wild mushrooms have antioxidant^16^, antitumor, and antimicrobial properties^17^. The nutraceuticals analyzed in these mushrooms are dietary fibres, polyunsaturated fatty acids, immunomodulatory proteins, polysaccharides, amino acids, keto acids, minerals, antioxidative vitamins, and other antioxidants^18^,^19^,^20^.

The genera *Ramaria* (Basidiomycetes, Agaricimycetes, Gomphales) and *Clavaria* (Basidiomycetes, Agaricimycetes, Agaricales) are worldwide in distribution. Some of the species of both these genera are used by people of native places for culinary purposes. Most of the species are not studied for detailed profiling of nutritional and nutraceutical compounds. The Northwestern Himalayan regions of India include the states of Himachal Pradesh (30°22 to 33°12 N latitude and 75°45 to 79°04 E longitude), Uttarakhand (28°43 N to 31°28 N latitude and 77°34 E to 81°03 E longitude), and some parts of Jammu and Kashmir (34°8 N and 77°34 E). The regions in these states have extensive areas under forest and hidden diversity of coral mushrooms. Few species of these coral mushrooms are used by local inhabitants for culinary purposes from ancient times while exact composition and nutraceutical utility of many species is still unknown to till date. There are very few reports about the exploration of scattered species of these genera from Northwestern Himalayas. The knowledge of culinary status of these species is restricted to aged people. During the frequent surveys to Northwestern Himalayas, six species of coral mushrooms belonging to genus *Ramaria* viz., *R. botrytis, R. rubripermanens, R. flava, R. flavescens, R. aurea, R. stricta* and six species belonging to genus *Clavaria* viz., *C. fragilis, C. coralloides,* C. *purpurea, C. vermicularis, C. amoena* and *C. rosea* were collected. Informations about their culinary status were collected from local inhabitants. All the species were studied for their detailed biochemical profiling and biological activities for the first time.

## Results and Discussion

### Nutrients Profiling

Among the twelve coral mushrooms, some coral mushrooms viz., *Ramaria botrytis, R. flava, R. flavescens, R. stricta, Clavaria fragilis* and *C. vermicularis* are traditionally used by the native people of Northwestern Himalayan regions for culinary purposes from ancient times (Table 1). The detailed composition of nutrients of coral mushrooms from Northwestern Himalayas is presented in Table 2. Protein contents in all the species varied between 10.81 ± 1.2–21.65 ± 0.2%. Protein contents of *Ramaria botrytis* and *Clavaria fragilis* were found to be higher than other species. Minimum percentage of protein contents was documented in *R. stricta* (10.81 ± 1.2%). All the species of coral mushrooms contained low percentage of fat ranged from 0.22–1.49%. *Ramaria botrytis* contained least percentage of crude fat (0.22%) and *R. flava* contained highest percentage of fat content (1.49%) in all the tweleve species. Coral mushrooms belonging to genus *Clavaria* contained lower percentage of fat as compared to *Ramaria* species. In general coral mushrooms of both genera from Northwestern Himalayas were found to be higher in protein and low in fat. However differences were observed in net values of individual species. Crude fibres ranged from 0.28–1.33% in all the species with highest percentage in *Ramaria botrytis* and lowest in *R. stricta*. Ash content varied between 0.23–1.27%. Carbohydrates were found to be in higher percentage as compared to other nutritional components and their percentage was ranged from 40.50–50.26%. Amongst twelve species of *Ramaria* and *Clavaria, Ramaria stricta* contained minimum percentages of all the nutrients analyzed. Nutrient contents of *Ramaria stricta* were found to be in lesser amount as compared to other species. Six minerals were analyzed from these species viz., Fe, Na, K, Ca, Mg and Cu. *Ramaria botrytis* and *Clavaria fragilis* contained higher amounts of the minerals and least values for these were detected in *Ramaria stricta*. All the species were found to be rich in Mg. Its highest amount was detected in *Ramaria botrytis* 15.7 ± 3.12 mg/100 g and lowest amount in *R. stricta* 8.4 ± 2.15 mg/100 g. Results obtained for preliminary studies to check the toxicity level due to presence of heavy metals showed negative results for all the species. This means that the tested species of Himalayan coral mushrooms do not contain any heavy metals which cause toxicity in many wild edible mushroom species due to the fact that mushrooms accumulate toxic heavy metals.

All the species contained glucose and rhamnose as the principal sugars. However, other sugars viz., xylose, mannose, galactose and fructose were also detected in trace amounts from all the species. *Ramaria stricta* contained lowest percentage of glucose (50.12 ± 2.54%) whereas *R. botrytis* contained highest percentage of glucose (69.11 ± 3.9%). There are previous reports on documentation of culinary edible species from native regions of Northwestern Himalayas but there are no reports on the detailed chemical and bioactive profiling of present species of coral mushrooms. Nutrients content of *Ramaria botrytis* were documented similar to the previously analyzed wild edible *R. brevispora* (21.1%), *R. formosa* (20.3%). But higher than wild culinary and common edible mushroom *Lyophyllum decastes* (18.31%)^21^. Moreover, nutrient profiling of presently tested species are very much similar to the composition of several comercially cultivated edible and highly prized medicinal mushroom species analyzed from Himalayan regions viz., *Clavaria cinerea, Agaricus bisporus, Boletus edulis, Morchella esculenta, Cordyceps sinensis*, and *Lentinula edod*^22^,^23^,^24^,^25^,^26^. Results obtained for nutritional composition of presently evaluated species are in confirmity with other species of wild and commercially cultivated mushrooms with high protein and carbohydrate contents and low fat levels which directly making them nutritionally valuable^27^. Although the nutrional compositions of all the twelve species differed but the values are significantly higher than previously worked out species of related genera viz., *Clavaria rosea, Agaricus arvensis, Lepiota leucothites, Amanita caesarea, Gymnopilus junonius, Coprinus atramentarius, Hygrocybe coccinea, Hygrophorus pustulatus*, and *Lactarius* pubescen collected from these regions^28^. Presence of small percentage of crude fibres is making these coral fungi important in nutritional point of view. Only few species viz., *Ramaria brevispora* and *Clavaria rosea* have been analyzed for the nutritional composition from Indian regions^21^. The results for the nutritional analysis of these species are comparable with previously documented species of medicinal mushrooms belonging to related and different genera from Northwestern Himalayas^29^.

### Bioactive Profiling

The detailed fatty acid composition of all the twelve species is presented in Table 3. The major fatty acids found in all the coral mushrooms were linoleic acid (C18:2), followed by Paullinic acid (C20:1) and palmitic acid (C16:0). Polyunsaturated fatty acids were the main group of fatty acids documented in all the species. *Ramaria botrytis* and *Clavaria fragilis* contained lower percentages of saturated fatty acids and higher polyunsaturated fatty acids as compared to other species. C18:2 trans-linoleic acid percentages in presently evaluated coral mushrooms was documented much higher than wild edible *Pleurotus ostreatus, Lactarius salmonicolor, Flammulina velutipes, Russula anthracina* and *Boletus reticulatus*^30^. In all the twelve species of coral mushrooms unsaturated fatty acids were predominated over satutrated fatty acids. Unsaturted fatty acids were ranged from 65–70% of total fatty acids.

Amino acids profiling showed the presence of eleven amino acids (essential and non essential) viz., aspartic acid, arginine, alanine, proline, tyrosine, valine, leucine, lysine, isoleucine, methionine and glutamic acid. All the amino acids were detected in appreciable amounts from all the species of coral mushrooms. However, in some species of coral mushrooms belonging to genus *Clavaria* amino acids viz., proline, lysine and isoleucine were not detected. The details of the amino acid profile are presented in Table 4. Glutamic acid was documented in higher percentage as comparted to other amino acids. The higher percentage of this was documented from *Ramaria botrytis* (3.17%) and *Clavaria fragilis* (3.15%).

Tocopherol contents in all the species are detailed in Table 5. α-tocopherol and β-tocopherol were found to be present in all the species. However, α-tocopherol was not documented in *Ramaria aurea* and *R. stricta*. Tocopherol contents were ranged from 0.01–1.71 μg/g in all the species. *Ramaria botrytis* and *Clavaria fragilis* contained maximum amount of all the three isomers as compared to other species. Results obtained for β-carotene, lycopene, phenolic compounds, ascorbic acid, and anthocyanidins composition of all the species is presented in Table 5. β-carotene in all the species ranged from 0.55–0.92 μg/100 g. Highest amount of this was detected in *Ramaria botrytis* (0.92 μg/100 g) and lowest in *R. stricta* (0.57 μg/100 g). Lycopene content in all the species ranged from 0.28–0.49 μg/100 g. Highest amount of this was detected in *R. botrytis* (0.49 g/100 g) and lowest in *R. stricta* (0.28 μg/100 g). Phenols were detected in significant amounts from all the species (40.32–56.35 mg/g). The highest amounts of phenolic compounds were detected in *R. botrytis* (56.35 mg/g) and lowest in *R. stricta* (40.32 mg/g). Ascorbic acid content of all the species ranged from (0.42–0.89 mg/100 g). Anthocyanidins ranged from 13.11–22.92 mg/100 g in all the twelve species. Highest content of these were detected in *Ramaria botrytis* (22.92 mg cyanidin chloride/100 g extract) and least in *R. stricta* (13.11 mg cyanidin chloride/100 g extract). Each species differed in net amounts of all these components with other species. High percentage of UFA showed the medicinal importance of these coral mushrooms as UFA increase the HDL cholesterol and decrease LDL cholesterol, triacyl-glycerol, lipid oxidation, and LDL susceptibility to oxidation^31^. All the three isomers viz., α- tocopherol, β-tocopherol and γ-tocopherol were documented from all the twelve species of coral mushrooms. However γ-tocopherol was not detected in four species of coral mushrooms viz., *Ramaria aurea, R. stricta, Clavaria amoena* and *C. rosea*. β-tocopherol was detected in higher amount as compared to α- tocopherol and γ tocopherol from all species of coral mushrooms. The composition of studied coral mushrooms is comparable to previously analyzed edible mushrooms reported from Northwestern Himalayas^27^. *R. botrytis* and *Clavaria fragilis* contained significantly higher amounts of tocopherol as compared to other species. The high levels of these two compounds correspond with higher oxidative activities and play a vital role in human body as α-tocopherol was considered the most active form of vitamin E in humans and it was reported to exhibit the highest biological activities^32^. All the coral mushrooms contained phenolic compounds in higher amounts than other bioactive compounds. Phenolic compounds of *Clavaria* and *Ramaria* analyzed presently were documented in much higher amount than *Clavaria* and *Ramaria* species evaluated from the forests of Western Ghats in Souther part of India^33^.

Presence of high phenolic compounds accounts for the high antioxidant properties of all these species^28^. Besides, their direct antioxidant activities, phenolic compounds are capable to promote other activities like antiproliferation, cell cycle regulation, and induction of apoptosis. Other bioactive compounds viz., β-carotene, lycopene, and ascorbic acids were detected in low amounts. Traces of anthocyanidins were also detected from all these species. The presence of these functional medicinal compounds in all the studied coral mushrooms is making them important for their pharmaceutical uses.

### Biological Activities

#### Antioxidant and Antibacterial activities

Antioxidant properties of all the species were expressed as EC_50_ values. The details are presented in Table 6. Stablities of DPPH radicals are widely used to evaluate the antioxidant activities of proton-donating substances according to their hydrogen-donating ability. DPPH radicals accept electrons or hydrogen radicals to form stable diamagnetic molecules. Higher EC_50_ values indicate the lower effectiveness for antioxidant properties and *vice versa*. EC_50_ values obtained for DPPH radical scavenging activity in all the twelve species showed differences in effectiveness. Amongst all, the species *Ramaria botrytis* and *Clavaria fragilis* showed lowest EC_50_ values and higher in *R. stricta. Ramaria botrytis* and *Clavaria fragilis* showed higher DPPH radical scavenging activity and *R. stricta* showed lower DPPH radical scavenging activities than other species. This could be explained by the DPPH radical-scavenging activity being related to the content of total phenolic compounds and total flavonoid contents found in the extracts. Similar results were documented for ABTS radical scavenging activities in which EC_50_ were ranged from 0.39–0.92 mg/mL. Lowest EC_50_ values were obtained for *Ramaria botrytis* and *Clavaria fragilis* showing high antioxidant activities of these species. Higher EC_50_ values were obtained for *Ramaria stricta* showing lowest ABTS radical scavenging activities of this species. Higher effectiveness in ferrous ion chelating activity and scavenging ability on nitric oxide was detected in *Ramaria botrytis* and low effectiveness was detected in *R. stricta.* In general, all the twelve coral mushrooms showed significant antioxidant properties measured on the basis of EC_50_ values. Each species showed different antioxidant activities with highly effective and less effective EC_50_ values. Better antioxidant properties of *Ramaria botrytis* and *Clavaria fragilis* are due to presence of higher phenolic compounds, β-carotene, lycopene, ascorbic acids, anthocyanidins, and tocopherol amounts in them which break the free radical chain by donating an electron to stabilize and terminate radical chain reactions or by showing pro-oxidant by maintaining the transition metal ions, Fe^3+^ and Cu^2+^ in their reduced forms. High reducing power of some species might be due to the presence of higher amounts of reducers in them. Role of antioxidants present in coral mushrooms can demonstrate their protective properties via two main types of antioxidants, namely, primary (chain breaking, free radical scavengers) and secondary or preventive. Secondary antioxidants are the consequence of deactivation of metals, inhibition or breakdown of lipid hydroperoxides, regeneration of primary antioxidants^34^,^35^.

The substances present in these mushrooms exhibit antioxidant activity by acting as inducers or promoting cell signals, leading to changes in gene expression, which result in the activation of enzymes that eliminate reactive oxigen species.

All the species showed antimicrobial activities interms of inhibition zones produced and MIC values against all the human pathogenic bacteria tested (Tables 7 and 8). The minimum inhibition concentration against all the pathogenic bacteria was ranged from 40 to 100 mg/mL for all the species. MIC values for *E. coli* showed 80, 100, 60, 80, 100 by *Ramaria botrytis, R. rubripermanens, R. flava R. flavescens R. aurea* and *R. stricta*, respectively and 80, 80, 40, 60, 80, 100 by *Clavaria fragilis, C. coralloides, C. purpurea C. vermicularis C. amoena* and *C. rosea*, respectively. The MIC values against other pathogenic bateria are deailed in Table 8. The screening of antibacterial activities of all the species indicates that inhibition zones produced by *Ramaria botrytis* and *Clavaria fragilis* are larger as compared to other species. As evident from the tables, the antimicrobial spectrum was found to be directly linked with concentrations of the coral mushroom extracts. In genearl, all the species showed broad spectrum against all the pathogenic microorganisms at higher concentrations. Maximum activities were observed against *E. coli* by *Ramaria botrytis* (11.1 ± 1.9 mm). The results obtained for broad spectrum under higher concentrations are similar as obtained for other widely used medicinal species of mushrooms^36^,^37^,^38^. Extracts from all the species showed activities against both gram positive and gram negative strains. The sensitivity of gram-positive bacteria to the mushroom extracts is in conformity with the previous studies on other medicinal and edible species due to the membrane composition of the bacterial stains^39^,^40^,^41^. The susceptibility of gram positive and gram negative bacteria to the mushroom extract might be due to the presence of low molecular weight or high molecular weight compounds as peptides and proteins in the mushrooms that allow the opening of the pores for ions transport and inhibit bacterial growth^42^,^43^. Difference in the antibacterial activities for different species is due to the production of different antimicrobial compounds by different species.

## Materials and Methods

### Collection and Processing of Samples

All the samples were collected during the frequent surveys to the different regions of Northwestern Himalayas. Informations about the culinary status were collected from native inhabitants. The species were taxonomicaly identified at Mushroom Research Centre, CSK Himachal Pradesh Agriculture University. Twelve species belonging to two genera *Ramaria* and *Clavaria* were subjected to detailed studies on biochemical profiling and their biological activities. The samples were vacuum dried and preserved in air-tight cellophane bags, with a small amount of 1-4-paradichlorobenzene in porous packets to keep them free of insects, for further analysis. Samples are deposited and available at Mushroom Research Centre (No. R501–R512).

### Chemical Profiling

For chemical profiling of nutrients, samples were powdered and analyzed for protein, fat, carbohydrates, ash, and crude fibres. Crude protein contents were estimated using the Kjeldahl method by calculating total nitrogen (N) and protein contents were expressed by N × 4.38^44^. Crude fat was estimated using a Soxhlet apparatus by extraction of powered samples with petroleum ether. Ash content was calculated by incineration in silica dishes at 525 ± 20 °C containing 5–10 g/sample. Fibre contents were estimated on de-fatted samples using the acid-alkali method (1.25% each). Total carbohydrates percentage was calculated by the difference as the total weight - (moisture content + protein content + crude fat + ash content + crude fibres). Minerals were analyzed using Atomic Absorption Spectophotometer (Perkin Elmer Analyst A 400, Waltham, MA, USA).

Preliminary tests were performed to check the presence of toxic metals. For this, diluted HCl (2%) and copper foil (1 × 1/2 cm strips) pretreated with concentrated HNO_3_ were taken. After that powered samples were acidified with 10–20 mL of 5 diluted HCl (2%) until colour changed from fairy pink to litmus. After this strips of copper foil were added and boiled for 30 min with addition of water from time to time to replace the losses by evaporation. The heavy metals got deposited on the copper foil and color was noted after 30 min. The color and results were interpreted for the presence of heavy metals.

For monosaccharides composition, samples were extracted with 70% aqueous methanol (2.5 mL and 1.5 mL). After this, the extracts were centrifuged at 4000 rpm (4 °C) for 10 min. Supernatants were collected and volumes were made up to 5 mL with 70% methanol. The extract was passed through Millipore filter (0.45 m) and injected to the HPLC^45^.

### Bioactive Profiling

#### Fatty Acid Composition

The samples were dissolved in 1 mL of solution (sodium hydroxide pellets (45 g) in 300 mL of 50% methanol and vortexed for 1 min. The solution was left for 5 minutes at 100 °C, vortexed again for 1 minute, and left at 100 °C in a water bath for 25 min. Methylation was done by adding 2 mL of solution (6 N hydrogen chloride in methanol) and vortexing for 1 min followed by heating (80 °C) in a water bath. For extraction of fatty acids, 1.25 mL of solution (25 mL methyl ter-butyl ether added to hexane) was added, and the solution was shaken for 10 min. Upper layer was removed and 3 mL of solution (10% sodium hydroxide in water while stirring) ws added. Finally, the top phase (2/3) was removed and transferred into a gas chromatography vial and injected. An Agilent 7890A gas chromatograph equipped with a flame ionization detector (FID) and fused silica column (25 m × 200 μm × 0.33 μm, Spelco, Sigma) was used in split mode of 40:1. The column temperature was programmed from 70 to 200 °C with 2 minutes hold at 70 °C, 6.5 rise/min and 5 minutes hold at 200 °C. The detector and injector temperature were 250 °C and 300 °C respectively. N_2_ and H_2_ were used as carrier and fuel gases, respectively.

#### Amino Acids

For amino acids profiling powdered samples (0.1 g) of all the species were extracted with 70% aqueous methanol (2.5 mL followed by 1.5 mL and 1 mL). Now the samples were centrifuged for 10 min (4000 rpm) at 4 °C. Supernatants were dissolved in aqueous methanol and the volume was made up to 5 mL. After that it was passed through Millipore filter (0.45 *μ*m). Samples were dried using vacuum oven and, to these dried samples, 20 *μ*L derivatising agent (prepared by ethanol: triethylamine: water: phenylisothiocaynate) was mixed and then re-dried. Now the samples were left for 25 min at room temperature. Finally, ethanol (1 mL) was added and injected into UPLC.

An Acquity UPLC from Waters India Pvt. Ltd. equipped with PDA (Photodiode array detector) and Pico. Tag column (3.9 × 150 mm) for amino acid analysis was used. Mobile phase A consisted of 0.1% TEA (triethylamine) in 940 ml water + 60 ml acetonitrile and mobile phase B consisted of 600 ml acetonitrile + 400 ml distilled water were used. Column temperature was 38 °C and amino acids were estimated at 254 nm. Flow rate was set as follow:


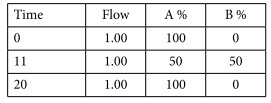


#### Tocopherol Composition

Tocopherols were estimated using HPLC following standard methods^46^. Firstly, samples were mixed with butylated hydroxytoluene (BHT) in hexane (10 mg/mL; 100 *μ*L) and IS solution in hexane (*δ* tocopherol; 1.6 *μ*g/mL; 250 *μ*L). Now, samples (500 mg) were vortexed for 1 min with methanol (4 mL). The samples were again vortexed with hexane (4 mL). After this, 2 mL of saturated NaCl aqueous solution was added, and the mixture was vortexed (1 min), followed by centrifugation at 4000 g for 5 min and the upper layer was separated. The samples were again re-extracted twice with hexane. The extracts were then vacuum-dried and re-dissolved in hexane (1 mL), followed by dehydration with anhydrous sodium sulphate, then filtered and transferred into a dark injection vial, and analyzed by HPLC (Waters India Pvt. Ltd.). Chromatographic comparisons were made by authentic standards.

### Profiling of Other Bioactive Compounds

β-carotene and lycopene were estimated from powdered samples. Samples (∼5 g) were extracted with 100 mL of methanol at 25 °C (150 rpm) for 24 hours and filtered through Whatman no. 2 filter paper. The residues were again re-extracted with 2 additional 100 mL portions of methanol. These extracts were evaporated to dryness at 42 °C, then redissolved in methanol at a concentration of 50 mg/mL, and stored at 4 °C. The dried methanolic extracts (100 mg) were shaken vigorously with acetone/hexane mixture (4:6) for 1 minute and filtered. The absorbance of the filtrate was measured at 453, 505, and 663 nm^47^. *β*-carotene and lycopene content were estimated using the following equation: Lycopene (mg/100 mL) = (0.0458 × *A*663) + (0.372 × *A*505) − (0.0806 × *A*453) β-carotene (mg/100 mL) = (0.216 × *A*663) − (0.304 × *A*505) + (0.452 × *A*453).

Phenolic compounds were quantified using Folin and Ciocalteu’s phenol reagent. Briefly, powdered samples (1 mL) were mixed with Folin and Ciocalteu’s phenol reagent. Now, 1 mL of saturated sodium carbonate solution (3 mL) was added to the mixture, and the volume was adjusted with distilled water to 10 mL. The reaction was kept in the dark for 90 min after that absorbance was read at 725 nm. Gallic acid was used to calculate the standard curve (0.01–0.4 mM; *R*^2^ = 0.9999) and the results were expressed as milligrams of gallic acid equivalents per gram of extract^48^. Ascorbic acid contents were quantified by colorimetric assays. For quantification, standard ascorbic acid solution (5 mL L-ascorbic acid in 3% phosphoric acid) was added to 5 mL of phosphoric acid. A microburette was filled with dye, and the samples were titrated with the dye solution to a pink color, which persisted for 15 seconds. The dye factor (milligrams of ascorbic acid per milliliter of dye using formula: 0.5/titrate) was determined. Sample (10 g) was grounded in metaphosphoric acid, and the volume was increased up to 100 mL. It was titrated after filtration until a pink color appeared^38^. The amount of ascorbic acid was calculated with the use of the following equation: mg of ascorbic acid per100 g or mL = titrate × dye factor × vol. made aliquot of extract × wt. of sample × 100.

Anthocyanidins were quantified by using standard protocol with minor modifications^49^. Briefly, samples (0.7 g) were mixed with the solvent (mixture of 85 : 15 (v/v) of ethyl alcohol and hydrochloric acid 1.5 M) followed by ultrasonication for 15 min and filtration through Whatman filter paper no. 1. Standard solution was prepared with cyaniding chloride with a concentration of 5–15 μg/mL. The absorption was measured at 546 nm. The total quantity of anthocyandins expressed in g of cyaniding chloride/100 g extract.

### Bioactivities

#### Antioxidant Activities

DPPH scavenging activity was measured with adding DPPH (200 *μ*L) solution at different concentrations (2–10 mg/mL) to 0.05 mL of the samples dissolved in ethanol. An equal amount of ethanol was added to the control. Ascorbic acid was used as the control^50^. The absorbance was read after 20 min at 517 nm and the inhibition was calculated using the formula DPPH scavenging effect (%) = *A*_0_ − *A*_*P*_/*A*_0_ × 100, where *A*_0_ represented the absorbance of the control and *A*_*P*_ represented the absorbance in the presence of the sample.

ABTS radical scavenging activity was measured following standard protocol^51^. Samples were added to 4 mL of diluted ABTS^+^ solution (prepared by adding 7 mM of the ABTS stock solution to 2.45 mM potassium persulfate, kept in the dark, at room temperature, for 12–16 h before use). The solution was then diluted with 5 mM saline (pH 7.4) phosphate-buffered and absorbance was measured at 730 nm after 30 min. The ABTS radical scavenging activity was calculated as S% = (*A*_control_ − *A*_sample_/*A*_control_) × 100.

For reducing power estimation, samples were mixed with sodium phosphate buffer (pH 6.6), 1 mM FeSO_4_, and 1% potassium ferricyanide and incubated for 20 min at 50 °C. Now, trichloroacetic acid was added and the mixtures were centrifuged. Supernatant was mixed with an equal volume of water and 0.5 mL 0.1% FeCl_3_. The absorbance was measured at 700 nm^52^.

For calculating iron chelating activities, samples were mixed with 3.7 mL of ultrapure water, after that the mixtures were reacted with ferrous chloride (2 mmol/L, 0.1 mL) and ferrozine (5 mmol/L, 0.2 mL) for 20 min and the absorbance was read at 562 nm with using EDTA as control. The chelating activities were calculated using the formula chelating activity (%) = [(*A*_*b*_ − *A*_*s*_)] × 100, where *A*_*b*_ is the absorbance of the blank and *A*_*s*_ is the absorbance in the presence of the extract^53^.

The scavenging activities of superoxide anion radicals were measured following standard method^54^. Samples and Tris-HCl buffer (50.0 mM, pH 8.2,3 mL) were incubated in a water bath at 25 °C for 20 min and after this pyrogallic acid (5.0 mM, 0.4 mL) was added. HCl solution (8.0 M, 0.1 mL) was added to terminate the reaction after 4 min. The absorbance of the mixture was measured at 320 nm. The scavenging ability was calculated using the following formula: scavenging ability (%) = (1 − *A*_sample_/*A*_control_) × 100. Where *A*_control_ is the absorbance of control without the polysaccharide sample and *A*_sample_ is the absorbance in the presence of the polysaccharide sample.

For ferric reducing antioxidant power (FRAP) assay, FRAP reagent was prepared by mixing TPTZ (2.5 mL, 10 mM in 40 mM HCl), 25 mL of 300 mM acetate buffer, and 2.5 mL of FeCl_3_ · 6H_2_O. Thereafter, FRAP reagent (1.8 mL) was incubated at 30 °C in water bath for 10 min. Then, absorbance was read at 0 min (t0). After this, 100 *μ*L of sample extract or standard and 100 *μ*L of distilled water were added to the test tube, mixed, and incubated at 30 °C for 30 min. Then, the absorbance was taken at 593 nm (t30). Ferrous sulphate was used as standard^55^,^56^,^57^. FRAP activity was determined against a standard curve of ferrous sulphate and the values were expressed as μM Fe^2+^ equivalents per gram of extract and calculated using the following equation: FRAP value = Absorbance (sample + FRAP reagent) − Absorbance (FRAP reagent).

#### Antibactetrial Activities

Extracts of the coral mushrooms powdered samples were prepared to analyze the antibacterial activities. For extract preparation, 5 g of mushrooms were macerated twice with 99.9% ethanol (50 mL) at room temperature for 24 h and filtered. The residues were twice extracted by ultrasonically assisted extraction with 50 ml of ethanol at room temperature for 30 min. The combined extracts were evaporated to dryness under vacuum. Samples were re-dissolved for checking antibacterial effects. Minimal inhibitory concentration (MIC) of the extracts were tested for *Escherichia coli, Klebsiella pneumonia, Vivrio cholerae, Pseudomonas aeruginosa, Vibrio alginolyticus* and *Staphylococcus aureus*, The strains were procured from Microbial Type Culture Collection and Gene Bank, CSIR, India. For this, bacterial strains were individually inoculated in the nutrient broth and incubated at 37 °C for 24 h. Mueller Hinton agar (MHA) was prepared and autoclaved and poured in petriplates and incubated at 37 °C for 24 h. The 24 h old bacterial broth cultures were inoculated in the petridishes. The stock solutions of sample extracts were prepared at a concentration of 100 mg/mL. Sterile antimicrobial disc was impregnated with extracts of the four concentrations tested. Positive control disc containing tetracycline (1 mg/mL) and negative control as EDTA was taken. These impregnated discs were allowed to dry at laminar air flow chamber for 2 h, and were placed at the respective bacterial plates and incubated at 37 °C for 24 h. The diameter (mm) of the growth inhibition halos produced by the extracts was examined. Results were calculated by measuring the zone of inhibition in millimetres (mm)^58^. MIC was determined followed by the turbidimetric method^59^. A stock solution of 100 μg/mL^−1^ was prepared and diluted to obtain various ranges of concentrations from 20 to 100 μg/mL^−1^. 0.5 mL of each of the dilutions of different concentrations was transferred into sterile test tube containing 2.0 mL of nutrient broth. To the test tubes, 0.5 mL of test organism previously adjusted to a concentration of 10^5^ cells/mL^−1^ was then introduced. A set of test tubes containing broth alone was used as control. All the test tubes and control were then incubated at 37 °C for 24 h. After the period of incubation, the tubes were studied for visible signs of growth or turbidity. The lowest concentration coral mushroom extracts that inhibited the growth of bacteria was taken as the minimum inhibitory concentration. All assays were carried out in triplicates and the control test was carried out with the broth alone.

### Statistical Analysis

All experiments for chemical and bioactive analysis were performed three times and with three replicates, and the results were expressed as mean ± SD values of 3 observations. The results were analyzed using one-way analysis of variance (ANOVA) and *p* < 0.05 was considered significant, and SPSS software (SPSS Inc., Chicago, IL, USA, version 16) was used to calculate differences.

## Conclusions

Coral mushrooms of Northwestern Himalayas are very important because of their culinary credentials and use from ancient times by native inhabitants. There are no previous reports about the excat chemical composition and bioactivities of these important mushrooms from Northwestern Himalayas. Presently investigated species have been collected from the regions native to northwestern Himalayas and evaluated for the exact composition of bioactive compounds and their *in vitro* antioxidant and antimicrobial activities. Some species of coral mushrooms are used by local people for culinary purposes. But, their knowledge is restricted to the aged villagers of the regions and hence neglected for the commercial exploitations. All the species were found to be rich in protein, minerals and contained important nutraceuticals such as unsaturated fatty acids, phenolics, carotenoids, ascorbic acid, tocopherols, and anthocyanidins which can be useful for their commercial use for nutritional therapy. All the species are observed free from toxic heavy metals and health promoting contituents like unsaturated fatty acids, essential amino acids, carotenoids, phenolic compounds with advantage of the additive effects of antioxidant compounds. Extracts of these coral mushrooms from Northwestern Himalayas showed broad spectrum of inhibition against human pathogenic bacterias. The extracts can be used as antibacterial constituents against human pathogenic bacterial strains tested presently.

## Additional Information

**How to cite this article:** Kumar Sharma, S. and Gautam, N. Chemical and Bioactive Profiling, and Biological Activities of Coral Fungi from Northwestern Himalayas. *Sci. Rep.* **7**, 46570; doi: 10.1038/srep46570 (2017).

**Publisher's note:** Springer Nature remains neutral with regard to jurisdictional claims in published maps and institutional affiliations.

## Figures and Tables

**Table 1:** Details of collections and culinary status of coral mushroom species collected from Northwestern Himalayas.

Species	Location	Altitude (m)	Culinary Status
*Ramaria botrytis*	Kalatope (Himachal Pradesh, India)	2300	Edible: Used by native people for culinary purposes
*R. rubripermanens*	Sonmarg (Jammu Kashmir, India)	2800	Unknown: People are not aware about culinary potential
*R. flava*	Janjehli (Himachal Pradesh, India)	2400	Edible: Used by native people for culinary purposes
*R. flavescens*	Macleodganj (Himachal Pradesh, India)	2200	Edible: Used by native people for culinary purposes
*R. aurea*	Nainital (Uttrakahnd, India)	2600	Unknown: People are not aware about culinary potential
*R. stricta*	Kalatope (Himachal Pradesh, India)	2300	Edible: Used by native people for culinary purposes
*Clavaria fragilis*	Janjehli (Himachal Pradesh, India)	2400	Edible: Used by native people for culinary purposes
*C. coralloides*	Kumaoon (Uttrakahnd, India)	2800	Unknown: People are not aware about culinary potential
*C. purpurea*	Macleodganj (Himachal Pradesh, India)	2200	Unknown: People are not aware about culinary potential
*C. vermicularis*	Sonmarg (Jammu Kashmir, India)	2800	Edible: Used by native people for culinary purposes
*C. amoena*	Nainital (Uttrakahnd, India)	2600	Unknown: People are not aware about culinary potential
*C. rosea*	Nainital (Uttrakahnd, India)	2600	Unknown: People are not aware about culinary potential

**Table 2:** Nutritional profiling of coral mushroom species collected from Northwestern Himalayas.

Nutritional profiling	*R. botrytis*	*R. rubripermanens*	*R. flava*	*R. flavescens*	*R. aurea*	*R. stricta*	*Clavaria fragilis*	*C. coralloides*	*C. purpurea*	*C. vermicularis*	*C. amoena*	*C. rosea*
**Nutrients (%)**												
Protein	21.65 ± 0.2^b^	16.32 ± 3.2^b^	15.80 ± 1.2^b^	14.60 ± 0.2^b^	13.30 ± 3.2^b^	10.81 ± 1.2^b^	18.21 ± 1.0^b^	11.95 ± 1.5^b^	10.15 ± 1.3^b^	11.75 ± 1.2^b^	12.80 ± 1.0^b^	13.61 ± 1.2^b^
Crude fat	0.22 ± 0.0^a^	1.49 ± 0.0^a^	1.26 ± 0.0^a^	1.18 ± 0.0^a^	0.91 ± 0.0^a^	0.37 ± 0.0^a^	0.27 ± 0.0^a^	0.39 ± 0.0^a^	0.44 ± 0.0^a^	0.38 ± 0.0^a^	0.44 ± 0.0^a^	0.41 ± 0.0^a^
Fibres	1.33 ± 0.0^a^	1.06 ± 0.0^a^	1.02 ± 0.2^a^	0.90 ± 0.0^a^	0.82 ± 0.0^a^	0.28 ± 0.0^a^	1.18 ± 0.0^a^	0.38 ± 0.0^a^	0.32 ± 0.0^a^	0.33 ± 0.0^a^	0.38 ± 0.0^a^	0.25 ± 0.0^a^
Ash	1.25 ± 0.0^a^	1.11 ± 0.0^a^	1.02 ± 0.2^a^	0.85 ± 0.0^a^	0.73 ± 0.0^a^	0.23 ± 0.0^a^	0.93 ± 0.0^a^	0.29 ± 0.0^a^	0.27 ± 0.0^a^	0.29 ± 0.0^a^	0.26 ± 0.0^a^	0.37 ± 0.0^a^
Carbohydrates	50.26 ± 1.9^c^	48.82 ± 2.5^c^	46.54 ± 2.9^c^	45.26 ± 1.9^c^	43.82 ± 2.5^c^	40.50 ± 2.9^c^	47.52 ± 1.9^c^	43.58 ± 2.9^c^	42.51 ± 2.8^c^	46.57 ± 2.9^c^	49.52 ± 2.7^c^	46.52 ± 2.9^c^
**Minerals (mg/100 g)**												
Fe	10.2 ± 0.02^a^	7.3 ± 0.08^b^	6.0 ± 0.01^a^	5.1 ± 0.01^b^	4.2 ± 0.02^b^	3.8 ± 0.08^a^	6.8 ± 0.2^a^	4.6 ± 0.02^a^	42 ± 0.8^a^	3.9 ± 0.08^a^	4.1 ± 0.08^a^	3.9 ± 0.0^a^
Na	1.4 ± 0.00^a^	1.02 ± 0.0^a^	0.95 ± 0.00^a^	0.93 ± 0.00^a^	0.82 ± 0.00^a^	0.72 ± 0.0^a^	0.82 ± 0.0^a^	0.69 ± 0.0^a^	0.76 ± 0.0^a^	0.70 ± 0.0^a^	0.70 ± 0.0^a^	0.79 ± 0.0^a^
K	2.3 ± 0.10^a^	1.3 ± 0.10^a^	0.94 ± 0.10^a^	0.80 ± 0.10^a^	0.72 ± 0.10^a^	0.63 ± 0.10^a^	0.75 ± 0.10^a^	0.67 ± 0. 0^a^	0.61 ± 0.10^a^	0.62 ± 0.10^a^	0.62 ± 0.0^a^	0.62 ± 0.0^a^
Ca	8.6 ± 1.10^b^	7.3 ± 1.12^b^	6.2 ± 1.10^b^	5.2 ± 1.10^b^	4.6 ± 1.10^b^	3.8 ± 1.12^b^	7.8 ± 1.4^b^	3.9 ± 1.1^b^	4.2 ± 1.11^b^	2.8 ± 1.12^b^	3.5 ± 1.42^b^	2.88 ± 1.12^b^
Mg	15.7 ± 3.12^b^	13.4 ± 2.10^b^	11.2 ± 3.10^b^	10.2 ± 3.10^b^	9.2 ± 1.15^b^	8.4 ± 2.15^b^	11.2 ± 2.8^b^	7.6 ± 2.15^b^	6.7 ± 2.10^b^	7.4 ± 2.15^b^	6.3 ± 2.10^b^	7.4 ± 2.10^b^
Cu	2.4 ± 0.04^a^	1.93 ± 0.02^a^	1.75 ± 0.04^a^	1.65 ± 0.04^a^	1.40 ± 0.04^a^	1.32 ± 0.02^a^	1.43 ± 0.0^a^	1.66 ± 0.0^a^	1.42 ± 0.0^a^	1.39 ± 0.02^a^	1.30 ± 0.0^a^	1.42 ± 0.0^a^
**Sugars (%)**												
Xylose	17.10 ± 0.3^b^	16.11 ± 1.0^b^	12.10 ± 1.7^a^	11.23 ± 0.3^b^	10.83 ± 1.2^b^	7.2 ± 0.00^b^	14.5 ± 1.2^b^	11.4 ± 1.2^b^	10.2 ± 0.00^b^	9.2 ± 1.1^b^	11.4 ± 0.00^b^	6.9 ± 0.0^b^
Glucose	69.11 ± 3.9^d^	62.09 ± 2.1^d^	57.60 ± 1.12^d^	55.19 ± 2.9^d^	52.69 ± 2.8^d^	50.12 ± 2.5^d^	57.12 ± 2.5^d^	51.11 ± 3.3^d^	48.11 ± 3.7^d^	43.19 ± 4.2^d^	47.11 ± 2.8^d^	47.10 ± 2.5^d^
Rhamnose	29.15 ± 2.7^c^	27.30 ± 1.3^c^	24.10 ± 1.5^c^	22.15 ± 2.7^c^	21.38 ± 1.9^c^	19.3 ± 1.10^b^	23.3 ± 2.10^b^	21.7 ± 1.15^b^	20.2 ± 2.1^b^	17.2 ± 1.4^b^	21.3 ± 3.14^b^	16.3 ± 1.2^b^
Mannose	4.75 ± 0.7^a^	3.98 ± 0.2^a^	2.99 ± 0.4^a^	2.67 ± 0.7^a^	1.98 ± 0.4^a^	1.23 ± 0.12^b^	2.21 ± 0.42^b^	1.55 ± 0.0^b^	1.23 ± 0.12^b^	1.31 ± 0.11^b^	1.53 ± 0.0^b^	1.43 ± 0.1^b^
Galactose	0.9 ± 0.0^a^	0.8 ± 0.0^a^	0.69 ± 0.0^a^	0.58 ± 0.0^a^	0.47 ± 0.0^b^	0.40 ± 2.10^b^	0.5 ± 2.30^b^	0.49 ± 0.0^b^	0.40 ± 2.10^b^	0.42 ± 0.0^b^	0.46 ± 0.0^b^	0.42 ± 0.0^a^
Fructose	0.7 ± 0.0^a^	0.8 ± 0.0^a^	0.7 ± 0.0^a^	0.38 ± 0.0^a^	0.30 ± 0.0^a^	0.23 ± 0.0^a^	0.5 ± 0.0^a^	0.46 ± 0.0^a^	0.43 ± 0.0^a^	0.41 ± 0.0^a^	0.39 ± 0.0^a^	0.37 ± 0.0^a^

Values are expressed as mean ± SE and different letters represent the significant difference in each column with ≤0.05 according to Tukey’s test.

**Table 3:** Fatty acids (%) profiling of coral mushroom species collected from Northwestern Himalayas.

Fatty acids	*Ramaria botrytis*	*R. rubripermanens*	Ramaria flava	Ramaria flavescens	R. aurea	R. stricta	*Clavaria* fragilis	*C.* coralloides	*C*. purpurea	*C.* vermicularis	*C.* amoena	*C. rosea*
C9:00	0.09 ± 0.0^a^	0.082 ± 0.0^a^	0.075 ± 0.0^a^	0.072 ± 0.0^a^	0.054 ± 0.0^a^	0.043 ± 0.0^a^	0.08 ± 0.0^a^	0.06 ± 0.0^a^	0.06 ± 0.0^a^	0.06 ± 0.0^a^	0.03 ± 0.0^a^	0.02 ± 0.0^a^
C10:0	0.47 ± 0.01^a^	0.36 ± 0.02^a^	0.32 ± 0.0^a^	0.30 ± 0.01^a^	0.26 ± 0.02^a^	0.22 ± 0.0^a^	0.46 ± 0.0^a^	0.41 ± 0.01^a^	0.42 ± 0.0^a^	0.43 ± 0.01^a^	0.47 ± 0.01^a^	0.39 ± 0.0^a^
C12:0	0.38 ± 0.0^a^	0.27 ± 0.0^a^	ND	0.38 ± 0.0^a^	0.07 ± 0.0^a^	ND	0.38 ± 0.0^a^	0.32 ± 0.0^a^	0.36 ± 0.0^a^	0.35 ± 0.0^a^	0.23 ± 0.0^a^	0.30 ± 0.0^a^
C16:0	2.19 ± 0.03^b^	2.11 ± 0.02^b^	1.95 ± 0.0b	1.82 ± 0.03^b^	1.69 ± 0.02^b^	1.5 ± 0.02^b^	2.13 ± 0.03^b^	1.98 ± 0.03^b^	1.89 ± 0.0^b^	1.78 ± 0.03^b^	1.19 ± 0.03^b^	1.68 ± 0.03^b^
C161:1	1.11 ± 0.0^a^	1.09 ± 0.0^a^	1.07 ± 0.0^a^	1.04 ± 0.0^a^	0.91 ± 0.0^a^	0.91 ± 0.0^a^	1.10 ± 0.0^a^	1.05 ± 0.0^a^	1.01 ± 0.0^a^	0.99 ± 0.0^a^	0.88 ± 0.0^a^	0.89 ± 0.0^a^
C17:1	0.49 ± 0.0^a^	0.42 ± 0.0^a^	0.37 ± 0.0^a^	0.29 ± 0.0^a^	0.22 ± 0.0^a^	0.17 ± 0.0^a^	0.47 ± 0.0^a^	0.41 ± 0.0^a^	0.43 ± 0.0^a^	0.40 ± 0.0^a^	0.36 ± 0.0^a^	0.37 ± 0.0^a^
C18:1	2.48 ± 0.0^b^	1.95 ± 0.0^b^	1.72 ± 0.0^a^	1.68 ± 0.0^a^	1.25 ± 0.0^a^	1.12 ± 0.0^a^	2.46 ± 0.0^b^	1.99 ± 0.0^b^	1.58 ± 0.0^a^	1.98 ± 0.0^b^	1.79 ± 0.0^a^	1.44 ± 0.0^a^
C18:2	8.28 ± 0.0^c^	6.54 ± 0.0^c^	5.79 ± 0.0^c^	5.28 ± 0.0^c^	4.94 ± 0.0^c^	3.93 ± 0.0^b^	8.18 ± 0.0^c^	7.18 ± 0.0^c^	7.23 ± 1.9^c^	7.68 ± 2.1^c^	7.12 ± 0.0^c^	6.28 ± 0.0^c^
C20:2	4.20 ± 0.2^a^	3.18 ± 0.3^a^	3.1 ± 0.1^b^	2.90 ± 0.2^b^	2.78 ± 0.3^b^	2.39 ± 0.0^b^	4.15 ± 0.0^b^	3.93 ± 0.2^b^	3.85 ± 0.0^b^	3.99 ± 0.2^b^	3.20 ± 0.2^b^	3.43 ± 0.2^b^
SFA	3.13 ± 0.2^b^	2.82 ± 0.1^b^	2.34 ± 0.1^b^	2.57 ± 0.2^b^	2.07 ± 0.3^b^	1.76 ± 0.1^a^	3.05 ± 0.3^b^	2.77 ± 0.3^b^	2.73 ± 0.2^b^	2.63 ± 0.2^b^	1.92 ± 0.2^a^	2.39 ± 0.2^b^
MUFA	4.08 ± 0.2^b^	3.46 ± 0.2^b^	3.16 ± 0.2^b^	3.01 ± 0.2^b^	2.38 ± 0.2^b^	2.20 ± 0.1^b^	4.03 ± 0.2^b^	3.45 ± 0.4^b^	3.02 ± 0.2^b^	3.37 ± 0.2^b^	3.03 ± 0.4^b^	2.7 ± 0.3^b^
PUFA	12.48 ± 1.1^d^	9.72 ± 0.8^b^	8.89 ± 0.9^b^	8.18 ± 0.8^b^	7.72 ± 0.8^b^	6.32 ± 0.9^b^	12.33 ± 1.3^d^	11.11 ± 1.5^d^	11.08 ± 1.1^d^	11.67 ± 1.1^d^	10.32 ± 1.1^d^	9.71 ± 1.1^b^

Values are expressed as mean ± SE and different letters represent the significant difference in each column with ≤ 0.05 according to Tukey’s test.

**Table 4:** Amino acid (%) profiling of coral mushroom species collected from Northwestern Himalayas.

Species	Aspartic acid	Arginine	Alanine	Proline	Tyrosine	Valine	Leucine	Lysine	Isoleucine	Methionine	Glutamic acid
*Ramaria botrytis*	0.44 ± 0.0^a^	0.34 ± 0.0^a^	0.19 ± 0.0^a^	0.12 ± 0.0^a^	0.29 ± 0.0^a^	0.87 ± 0.0^a^	1.17 ± 0.0^a^	0.88 ± 0.0^a^	0.64 ± 0.0^a^	0.33 ± 0.0^a^	3.17 ± 0.0^b^
*R. rubripermanens*	0.42 ± 0.0^a^	0.31 ± 0.0^a^	0.17 ± 0.0^a^	0.09 ± 0.0^a^	0.27 ± 0.0^a^	0.85 ± 0.0^a^	0.97 ± 0.0^a^	0.81 ± 0.0^e^	0.63 ± 0.0^a^	0.31 ± 0.0^a^	3.01 ± 0.0^b^
*R. flava.*	0.25 ± 0.0^a^	0.26 ± 0.0^a^	0.10 ± 0.0^c^	0.07 ± 0.0^a^	0.23 ± 0.0^a^	0.80 ± 0.0^a^	0.94 ± 0.0^a^	0.83 ± 0.0^a^	0.60 ± 0.0^a^	0.30 ± 0.0^a^	2.98 ± 0.0^b^
*R. flavescens*	0.39 ± 0.0^a^	0.33 ± 0.0^a^	0.16 ± 0.0^a^	0.08 ± 0.0^a^	0.25 ± 0.0^a^	0.75 ± 0.0^a^	0.89 ± 0.0^a^	0.80 ± 0.0^a^	0.62 ± 0.0^a^	0.32 ± 0.0^a^	2.87 ± 0.0^b^
*R. aurea*	0.38 ± 0.0^a^	0.29 ± 0.0^a^	0.14 ± 0.0^a^	0.06 ± 0.0^a^	0.28 ± 0.0^a^	0.69 ± 0.0^a^	0.83 ± 0.0^a^	0.82 ± 0.0^a^	0.58 ± 0.0^a^	0.28 ± 0.0^a^	2.72 ± 0.0^b^
*R. stricta*	0.34 ± 0.0^a^	0.27 ± 0.0^a^	0.13 ± 0.0^a^	0.02 ± 0.0^a^	0.21 ± 0.0^a^	0.63 ± 0.0^a^	0.73 ± 0.0^a^	0.72 ± 0.0^a^	0.52 ± 0.0^a^	ND	2.16 ± 0.0^b^
*Clavaria fragilis*	0.42 ± 0.0^a^	0.33 ± 0.0^a^	0.18 ± 0.0^a^	0.11 ± 0.0^a^	0.28 ± 0.0^a^	0.85 ± 0.0^a^	1.14 ± 0.0^a^	0.78 ± 0.0^a^	0.63 ± 0.0^a^	0.33 ± 0.0^a^	3.15 ± 0.0^b^
*C. coralloides*	0.39 ± 0.0^a^	0.29 ± 0.0^a^	0.15 ± 0.0^a^	0.07 ± 0.0^a^	0.26 ± 0.0^a^	0.80 ± 0.0^a^	0.99 ± 0.0^a^	0.79 ± 0.0^a^	ND	0.27 ± 0.0^a^	3.03 ± 0.0^b^
*C. purpurea*	0.36 ± 0.0^a^	0.26 ± 0.0^a^	0.17 ± 0.0^a^	ND	0.23 ± 0.0^a^	0.84 ± 0.0^a^	0.93 ± 0.0^a^	0.86 ± 0.0^a^	0.61 ± 0.0^a^	0.29 ± 0.0^a^	2.95 ± 0.0^b^
*C. vermicularis*	0.32 ± 0.0^a^	0.28 ± 0.0^a^	0.17 ± 0.0^a^	0.05 ± 0.0^a^	0.25 ± 0.0^a^	0.83 ± 0.0^a^	0.97 ± 0.0^a^	0.83 ± 0.0^a^	0.60 ± 0.0^a^	0.28 ± 0.0^a^	2.85 ± 0.0^b^
*C. amoena*	0.29 ± 0.0^a^	0.30 ± 0.0^a^	0.14 ± 0.0^a^	ND	0.29 ± 0.0^a^	0.81 ± 0.0^a^	0.92 ± 0.0^a^	ND	ND	0.26 ± 0.0^a^	2.76 ± 0.0^b^
*C. rosea*	0.27 ± 0.0^a^	0.32 ± 0.0^a^	0.16 ± 0.0^a^	0.06 ± 0.0^a^	0.26 ± 0.0^a^	0.82 ± 0.0^a^	0.88 ± 0.0^a^	0.77 ± 0.0^a^	0.59 ± 0.0^a^	0.27 ± 0.0^a^	2.62 ± 0.0^b^

Values are expressed as mean ± SE and different letters represent the significant difference in each column with ≤ 0.05 according to Tukey’s test ND = not detected.

**Table 5:** Bioactive profiling of coral mushroom species collected from Northwestern Himalayas.

Bioactive compounds	*Ramaria botrytis*	*R. rubripermanens*	R. flava	R. flavescens	R. aurea	R. stricta	*C.* fragilis	*C.* coralloides	*C.* purpurea	*C.* vermicularis	*C.* amoena	*C. rosea*
Phenolic compounds (mg/100 g of gallic acid)	56.35 ± 2.11^b^	53.10 ± 1.10^b^	45.32 ± 2.17^b^	43.11 ± 2.10^b^	40.32 ± 3.7^b^	33.11 ± 2.15^b^	55.18 ± 3.10^b^	52.19 ± 2.8^b^	44.39 ± 3.11^b^	42.57 ± 2.9^b^	41.65 ± 2.44^b^	34.10 ± 3.45^b^
Beta carotene (μg/100 g)	0.92 ± 0.0^a^	0.88 ± 0.0^a^	0.75 ± 0.0^a^	0.61 ± 0.0^a^	0.55 ± 0.0^a^	0.51 ± 0.0^a^	0.90 ± 0.0^a^	0.87 ± 0.0^a^	0.73 ± 0.0^a^	0.66 ± 0.0^a^	0.58 ± 0.0^a^	0.55 ± 0.0^a^
Lycopene (μg/100 g)	0.49 ± 0.0^a^	0.48 ± 0.0^a^	0.38 ± 0.0^a^	0.38 ± 0.0^a^	0.28 ± 0.0^a^	0.18 ± 0.0^a^	0.48 ± 0.0^a^	0.46 ± 0.0^a^	0.39 ± 0.0^a^	0.37 ± 0.0^a^	0.29 ± 0.0^a^	0.21 ± 0.0^a^
Ascorbic acid (mg/100 g)	0.89 ± 0.0^a^	0.86 ± 0.0^a^	0.52 ± 0.0^a^	0.49 ± 0.0^a^	0.42 ± 0.0^a^	0.33 ± 0.0^a^	0.87 ± 0.0^a^	0.84 ± 0.0^a^	0.59 ± 0.0^a^	0.45 ± 0.0^a^	0.44 ± 0.0^a^	0.39 ± 0.0^a^
Anthocyanins (mg cyanidin chloride/100 g extract)	22.92 ± 0.35^a^	17.09 ± 0.31^a^	15.12 ± 0.15^a^	14.21 ± 0.00^a^	13.11 ± 1.23^a^	11.21 ± 0.00^a^	21.95 ± 1.3^a^	18.04 ± 2.1^a^	16.19 ± 0.1^a^	15.29 ± 2.03^a^	13.94 ± 1.23^a^	12.44 ± 0.0^a^
α-tocopherol (mg/100 g)	0.004 ± 0.00^a^	0.003 ± 0.0^a^	0.003 ± 0.0^a^	0.001 ± 0.0^a^	0.50 ± 0.0^a^	0.42 ± 0.0^a^	0.003 ± 0.00^a^	0.002 ± 0.00^a^	0.002 ± 0.00^a^	0.001 ± 0.00^a^	0.52 ± 0.02^a^	0.44 ± 0.0^a^
β-tocopherol (mg/100 g)	0.076 ± 0.001^a^	0.072 ± 0.0^a^	0.068 ± 0.001^a^	0.062 ± 0.0^a^	0.060 ± 0.0^a^	0.052 ± 0.0^a^	0.074 ± 0.0^a^	0.071 ± 0.0^a^	0.069 ± 0.0^a^	0.065 ± 0.0^a^	0.063 ± 0.0^a^	0.052 ± 0.0^a^
γ-tocopherol (mg/100 g)	0.71 ± 0.02^a^	0.62 ± 0.0^a^	0.61 ± 0.0^a^	0.52 ± 0.0^a^	ND	ND	0.70 ± 0.0^a^	0.65 ± 0.0^a^	0.62 ± 0.0^a^	0.58 ± 0.0^a^	ND	ND

Values are expressed as mean ± SE and different letters represent the significant difference in each column with p ≤ 0.05 according to Tukey’s test. ND = not detected.

**Table 6:** EC50 values (mg/mL) exhibiting antioxidant activities of coral mushrooms from Northwestern Himalayas.

Species	DPPH radicals scavenging activity	ABTS radicals scavenging activity	Scavenging ability on superaxide anion radicals	Ferric reducing antioxidant power	Iron Chelating effect
*Ramaria botrytis*	0.68 ± 0.0^a^	0.39 ± 0.0^a^	0.86 ± 0.0^a^	0.92 ± 0.0^a^	1.12 ± 0.0^a^
*R. rubripermanens*	0.76 ± 0.0^a^	0.43 ± 0.0^a^	1.12 ± 0.1^a^	1.15 ± 0.0^a^	1.22 ± 0.0^a^
*R. flava.*	0.88 ± 0.0^a^	0.49 ± 0.0^a^	1.35 ± 0.0^a^	1.18 ± 0.0^a^	1.34 ± 0.0^a^
*R. flavescens*	1.52 ± 0.0^a^	0.52 ± 0.0^a^	1.48 ± 0.0^b^	1.22 ± 0.0^b^	1.38 ± 0.0^a^
*R. aurea*	1.58 ± 0.0^a^	0.59 ± 0.0^a^	1.55 ± 0.0^b^	1.28 ± 0.0^b^	1.44 ± 0.0^a^
*R. stricta*	1.72 ± 0.0^b^	0.92 ± 0.0^a^	1.58 ± 0.3^b^	1.32 ± 0.0^b^	1.58 ± 0.0^b^
*Clavaria fragilis*	0.69 ± 0.0^a^	0.40 ± 0.0^a^	0.88 ± 0.0^a^	0.93 ± 0.0^a^	1.14 ± 0.0^a^
*C. coralloides*	0.75 ± 0.0^a^	0.41 ± 0.0^a^	1.11 ± 0.0^a^	1.13 ± 0.0^a^	1.21 ± 0.0^a^
*C. purpurea*	0.86 ± 0.0^a^	0.47 ± 0.0^a^	1.30 ± 0.0^a^	1.16 ± 0.0^a^	1.32 ± 0.0^a^
*C. vermicularis*	1.48 ± 0.0^a^	0.50 ± 0.0^a^	1.44 ± 0.0^b^	1.20 ± 0.0^a^	1.34 ± 0.0^a^
*C. amoena*	1.52 ± 0.0^a^	0.56 ± 0.0^a^	1.50 ± 0.0^b^	1.26 ± 0.0^a^	1.40 ± 0.0^a^
*C. rosea*	1.68 ± 0.0^b^	0.89 ± 0.0^a^	1.53 ± 0.0^b^	1.30 ± 0.0^a^	1.48 ± 0.0^a^
Ascorbic acid	0.11 ± 0.0^a^	0.29 ± 0.0^a^	0.09 ± 0.0^a^	0.07 ± 0.0^a^	—
EDTA	—	—	—	—	0.27 ± 0.0^a^

Values are expressed as mean ± SE and different letters represent the significant difference in each column with p ≤ 0.05 according to Tukey’s test.

**Table 7:** Inhibition zone (mm) shown by coral mushroom species of Northwestern Himalayas against human pathogenic bacterial strains.

Species	Con. (%)	*Escherichia coli*	*Klebsiella pneumonia*	*Vivrio cholerae*	*Pseudomonas aeruginosa*	*Vibrio alginolyticus*	*Streptococcus pneumonia*	*Positive Control (Tetracycline*)	*Negative Control EDTA*
R. botrytis	25	6.2 ± 0.1^a^	—	—	2.2 ± 0.1^a^	—	—	10.91 ± 1.5^b^	—
	50	8.2 ± 0.3^a^	—	3.2 ± 0.2^a^	3.2 ± 0.3^a^	—	2.1 ± 0.2^a^	13.32 ± 1.1^b^	—
	75	10.2 ± 0.4^b^	4.2 ± 0.5^a^	4.3 ± 0.2^a^	5.2 ± 0.4^a^	2.2 ± 0.2^a^	3.3 ± 0.2^a^	17.23 ± 1.8^c^	—
	100	11.1 ± 1.9	5.3 ± 0.6^a^	5.1 ± 0.3^a^	7.1 ± 1.9^a^	3.3 ± 0.6^a^	4.1 ± 0.3^a^	18.92 ± 2.1^c^	—
R. rubripermanens	25	5.8 ± 0.2^a^	—	—	1.2 ± 0.0^a^	—	—	10.24 ± 1.3^b^	—
	50	7.8 ± 0.3^a^	—	1.2 ± 0.1^a^	2.7 ± 0.1^a^	—	1.2 ± 0.1^a^	13.19 ± 1.4^b^	—
	75	9.5 ± 0.4^a^	1.2 ± 0.5^a^	2.3 ± 0.2^a^	3.4 ± 0.2^a^	1.8 ± 0.1	2.5 ± 0.6^a^	16.21 ± 1.2^c^	—
	100	10.4 ± 1.2^b^	3.3 ± 0.6^a^	4.5 ± 0.8^a^	4.1 ± 1.0^a^	2.3 ± 0.4^a^	4.5 ± 0.8^a^	18.61 ± 2.3^c^	—
R. flava.	25	5.4 ± 0.2^a^	—	—	1.0 ± 0.0^a^	—	—	10.11 ± 2.5^b^	—
	50	7.2 ± 0.3^a^	—	—	2.5 ± 0.3^a^	—	—	13.54 ± 1.4^b^	—
	75	9.8 ± 1.4^a^	—	2.3 ± 0.1^a^	3.2 ± 0.2^a^	—	2.3 ± 0.1^a^	17.55 ± 1.7^c^	—
	100	10.1 ± 1.2^b^	2.3 ± 0.1^a^	3.5 ± 0.5^a^	3.4 ± 0.8^a^	1.9 ± 0.2^a^	3.4 ± 0.5^a^	18.37 ± 2.4^c^	—
R. flavescens	25	5.2 ± 0.8^a^	—	—	—	—	—	10.76 ± 1.2^b^	—
	50	6.9 ± 0.5^a^	—	—	2.2 ± 0.1^a^	—	—	12.32 ± 2.1^b^	—
	75	9.2 ± 1.3^a^	—	1.7 ± 0.2^a^	2.8 ± 0.4^a^	—	1.6 ± 0.2^a^	16.21 ± 1.3^c^	—
	100	10.2 ± 2.2^b^	1.1 ± 0.0^a^	2.6 ± 0.8^a^	3.2 ± 0.7^a^	1.4 ± 0.^a^	2.5 ± 0.8^a^	17.85 ± 2.1^c^	—
R. aurea	25	4.8 ± 0.5^a^	—	—	—	—	—	10.27 ± 1.2^b^	—
	50	6.4 ± 1.3^a^	—	—	—	—	—	13.38 ± 1.4^b^	—
	75	8.7 ± 1.4^a^	—	—	1.2 ± 0.0^a^	—	—	16.20 ± 2.1^c^	—
	100	9.9 ± 1.2^a^	—	2.9 ± 0.2^a^	2.5 ± 0.2^a^	1.1 ± 0.0^a^	2.4 ± 0.2^a^	17.82 ± 1.8^c^	—
R. stricta	25	4.4 ± 0.5^a^	—	—	—	—	—	10.21 ± 1.2^b^	
	50	6.2 ± 0.3^a^	—	—	—	—	—	13.22 ± 1.3^b^	—
	75	8.2 ± 0.4^a^	—	1.1 ± 0.0^a^	—	—	1.0 ± 0.0^a^	17.58 ± 1.3^c^	—
	100	9.1 ± 02^a^	—	2.1 ± 0.2^a^	1.0 ± 0.0^a^	—	2.2 ± 0.2^a^	18.26 ± 1.8^c^	—
*Clavaria fragilis*	25	6.1 ± 1.2^a^	—	—	2.3 ± 0.0^a^	—	—	10.91 ± 1.5^b^	—
	50	7.8 ± 1.3^a^	2.5 ± 0.2^a^	3.1 ± 0.4^a^	3.1 ± 0.3^a^	1.3 ± 0.1^a^	1.1 ± 0.0^a^	13.24 ± 1.6^b^	—
	75	9.8 ± 1.4^a^	3.7 ± 0.3^a^	4.1 ± 0.3^a^	4.9 ± 0.2^a^	2.3 ± 0.2^a^	2.2 ± 0.0^a^	15.23 ± 1.8^c^	—
	100	10.6 ± 1.2^b^	4.8 ± 0.4^a^	5.2 ± 0.1^a^	6.8 ± 1.3^a^	3.2 ± 0.0^a^	3.4 ± 0.0^a^	17.92 ± 2.2^c^	—
*C.* coralloides	25	4.5 ± 0.8^a^	—	—	1.3 ± 0.0^a^	—	—	10.20 ± 1.3^b^	—
	50	5.8 ± 0.7^a^	—	1.1 ± 0.0^a^	2.8 ± 0.1^a^	—	1.3 ± 0.2^a^	13.24 ± 1.5^b^	—
	75	7.4 ± 0.4^a^	1.1 ± 0.0^a^	2.7 ± 0.3^a^	3.5 ± 0.2^a^	1.9 ± 0.0^a^	2.6 ± 0.4^a^	17.20 ± 1.3^c^	—
	100	8.7 ± 1.5^a^	3.2 ± 0.2^a^	4.7 ± 0.9^a^	3.9 ± 1.3^a^	2.7 ± 0.3^a^	4.6 ± 0.3^a^	18.22 ± 2.2^c^	—
*C.* purpurea	25	5.5 ± 0.2^a^	—	—	1.2 ± 0.0^a^	—	—	10.75 ± 1.2^b^	—
	50	7.4 ± 0.7^a^	—	—	2.7 ± 0.3^a^	—	0.9 ± 0.0^a^	13.28 ± 1.4^b^	—
	75	8.9 ± 1.3^a^	1.2 ± 0.1^a^	2.4 ± 0.1^a^	3.4 ± 0.1^a^	1.1 ± 0.0^a^	2.5 ± 0.0^a^	16.20 ± 1.2^c^	—
	100	9.5 ± 1.0^a^	2.5 ± 0.4^a^	3.6 ± 0.5^a^	3.8 ± 0.1^a^	2.1 ± 0.5^a^	3.6 ± 0.8^a^	18.52 ± 2.2^c^	—
*C.* vermicularis	25	5.5 ± 0.4^a^	—	—	—	—	—	10.91 ± 1.5^b^	—
	50	7.3 ± 0.6^a^	—	—	2.4 ± 0.1^a^	—	—	13.32 ± 1.1^b^	—
	75	8.5 ± 1.6^a^	—	1.8 ± 0.0^a^	2.9 ± 0.4^a^	—	1.7 ± 0.2^a^	16.21 ± 1.2^c^	—
	100	9.9 ± 1.9^a^	1.0 ± 0.0^a^	2.9 ± 0.2^a^	3.5 ± 0.7^a^	1.5 ± 0.0^a^	2.3 ± 0.2^a^	17.90 ± 2.2^c^	—
*C.* amoena	25	4.9 ± 0.8^a^	—	—	—	—	—	10.21 ± 1.2^b^	—
	50	6.6 ± 1.2^a^	—	—	—	—	—	13.26 ± 1.4^b^	—
	75	8.9 ± 1.9^a^	—	—	1.2 ± 0.0^a^	—	—	17.15 ± 1.3^c^	—
	100	9.7 ± 1.8^a^	—	1.8 ± 0.1^a^	2.5 ± 0.2^a^	1.1 ± 0.0^a^	2.4 ± 0.2^a^	18.24 ± 2.2^c^	—
C. rosea	25	4.4 ± 0.5^a^	—	—	—	—	—	10.84 ± 1.2^b^	—
	50	6.2 ± 0.3^a^	—	—	—	—	—	13.24 ± 1.3^b^	—
	75	8.2 ± 0.4^a^	—	1.3 ± 0.0^a^	—	—	1.3 ± 0.0^a^	17.43 ± 1.3^c^	—
	100	9.1 ± 02^a^	—	2.2 ± 0.0^a^	1.1 ± 0.0^a^	—	2.5 ± 0.2^a^	18.91 ± 2.3^c^	—

Values are expressed as mean ± SE and different letters represent the significant difference in each column with p ≤ 0.05 according to Tukey’s test.

**Table 8:** MIC of studied coral mushroom species against tested pathogenic bacterial starins.

Species	Con. (%)	*Escherichia coli*	*Klebsiella pneumonia*	*Vivrio cholerae*	*Pseudomonas aeruginosa*	*Vibrio alginolyticus*	*Streptococcus pneumonia*
*R. botrytis*	20	**+++**	**+++**	**+++**	**+++**	**+++**	**+++**
	40	**++**	**++**	**++**	**++**	**++**	**++**
	60	**++**	**++**	**+**	**+**	**+**	**+**
	80	**+**	**+**	**+**	**+**	**+**	**+**
	100	**+**	**+**	**+**	**+**	**−**	**+**
*R. rubripermanens*	20	**+++**	**+++**	**+++**	**+++**	**+++**	**+++**
	40	**++**	**++**	**++**	**++**	**++**	**++**
	60	**++**	**++**	**++**	**++**	**++**	**++**
	80	**++**	**++**	**++**	**++**	**+**	**+**
	100	**+**	**+**	**+**	**+**	**+**	**-**
*R. flava.*	20	**+++**	**+++**	**+++**	**+++**	**+++**	**+++**
	40	**++**	**++**	**++**	**++**	**++**	**++**
	60	**+**	**++**	**++**	**++**	**++**	**++**
	80	**+**	**++**	**++**	**+**	**++**	**+**
	100	**+**	**+**	**+**	**+**	**+**	**-**
*R. flavescens*	20	**+++**	**+++**	**+++**	**+++**	**+++**	**+++**
	40	**++**	**++**	**++**	**+++**	**++**	**++**
	60	**++**	**++**	**++**	**++**	**++**	**++**
	80	**+**	**++**	**++**	**++**	**+**	**++**
	100	**+**	**+**	**+**	**++**	**+**	**+**
*R. aurea*	20	**+++**	**+++**	**+++**	**+++**	**+++**	**+++**
	40	**++**	**++**	**+++**	**++**	**++**	**++**
	60	**++**	**++**	**++**	**++**	**++**	**++**
	80	**++**	**++**	**++**	**++**	**++**	**+**
	100	**+**	**+**	**+**	**+**	**+**	**-**
*R. stricta*							
	20	**+++**	**+++**	**+++**	**+++**	**+++**	**+++**
	40	**+++**	**+++**	**+++**	**+++**	**+++**	**+++**
	60	**++**	**+++**	**++**	**++**	**++**	**++**
	80	**++**	**++**	**++**	**++**	**++**	**++**
	100	**++**	**++**	**++**	**++**	**+=**	**++**
						
*Clavaria fragilis*	20	**+++**	**+++**	**+++**	**+++**	**+++**	**+++**
	40	**++**	**++**	**++**	**++**	**++**	**++**
	60	**++**	**++**	**+**	**+**	**+**	**+**
	80	**+**	**+**	**+**	**+**	**+**	**+**
	100	**+**	**+**	**+**	**+**	**-**	**+**
	20	**+**	**+**	**+**	**+**	**+**	**+**
*C. coralloides*	20	**++**	**++**	**++**	**++**	**++**	**++**
	40	**++**	**++**	**++**	**++**	**++**	**++**
	60	**++**	**++**	**++**	**++**	**+**	**+**
	80	**+**	**+**	**+**	**+**	**+**	**-**
	100	**+**	**+**	**+**	**+**	**+**	**+**
*C. purpurea*	20	**++**	**++**	**++**	**++**	**++**	**++**
	40	**+**	**++**	**++**	**++**	**++**	**++**
	60	**+**	**++**	**++**	**+**	**++**	**+**
	80	**+**	**+**	**+**	**+**	**+**	**-**
	100	**+**	**+**	**+**	**+**	**+**	**+**
*C. vermicularis*	20	**++**	**++**	**++**	**+++**	**++**	**++**
	40	**++**	**++**	**++**	**++**	**++**	**++**
	60	**+**	**++**	**++**	**++**	**+**	**++**
	80	**+**	**+**	**+**	**++**	**+**	**+**
	100	**+**	**+**	**+**	**+**	**+**	**+**
*C. amoena*	20	**++**	**++**	**+++**	**++**	**++**	**++**
	40	**++**	**++**	**++**	**++**	**++**	**++**
	60	**++**	**++**	**++**	**++**	**++**	**+**
	80	**+**	**+**	**+**	**+**	**+**	**-**
	100	**+**	**+**	**+**	**+**	**+**	**+**
*C. rosea*	20	**+++**	**+++**	**+++**	**+++**	**+++**	**+++**
	40	**++**	**+++**	**++**	**++**	**++**	**++**
	60	**++**	**++**	**++**	**++**	**++**	**++**
	80	**++**	**++**	**++**	**++**	**+**	**++**
	100	**+**	**++**	**+**	**+**	**+**	**+**

*MIC concentration; −No growth; +Cloudy solution (slight growth); ++Turbid solution (strong growth); +++highly turbid solution (dense growth). Control readings = Nil for all the concentrations.
